# Inhibition of UBE2L6 attenuates ISGylation and impedes ATRA‐induced differentiation of leukemic cells

**DOI:** 10.1002/1878-0261.12614

**Published:** 2020-05-01

**Authors:** Nina Orfali, Deborah Shan‐Krauer, Tracey R. O’Donovan, Nigel P. Mongan, Lorraine J. Gudas, Mary R. Cahill, Mario P. Tschan, Sharon L. McKenna

**Affiliations:** ^1^ Cork Cancer Research Centre & Cancer Research at UCC University College Cork Ireland; ^2^ Department of Hematology Cork University Hospital Ireland; ^3^ Department of Pharmacology Weill Cornell Medical College New York NY USA; ^4^ Division of Experimental Pathology Institute of Pathology University of Bern Switzerland; ^5^ Faculty of Medicine and Health Science School of Veterinary Medicine and Science University of Nottingham UK

**Keywords:** AML, APL, ATRA, differentiation, ISG15, UBE2L6

## Abstract

Ubiquitin/ISG15‐conjugating enzyme E2L6 (UBE2L6) is a critical enzyme in ISGylation, a post‐translational protein modification that conjugates the ubiquitin‐like modifier, interferon‐stimulated gene 15 (ISG15), to target substrates. Previous gene expression studies in acute promyelocytic leukemia (APL) cells showed that all‐*trans*‐retinoic acid (ATRA) altered the expression of many genes, including UBE2L6 (200‐fold) and other members of the ISGylation pathway. Through gene expression analyses in a cohort of 98 acute myeloid leukemia (AML) patient samples and in primary neutrophils from healthy donors, we found that *UBE2L6* gene expression is reduced in primary AML cells compared with normal mature granulocytes. To assess whether UBE2L6 expression is important for leukemic cell differentiation—two cell line models were employed: the human APL cell line NB4 and its ATRA‐resistant NB4R counterpart, as well as the ATRA‐sensitive human AML HL60 cells along with their ATRA‐resistant subclone—HL60R. ATRA strongly induced UBE2L6 in NB4 APL cells and in ATRA‐sensitive HL60 AML cells, but not in the ATRA‐resistant NB4R and HL60R cells. Furthermore, short hairpin (sh)RNA‐mediated *UBE2L6* depletion in NB4 cells impeded ATRA‐mediated differentiation, suggesting a functional role for UBE2L6 in leukemic cell differentiation. In addition, ATRA induced *ISG15* gene expression in NB4 APL cells, leading to increased levels of both free ISG15 protein and ISG15 conjugates. UBE2L6 depletion attenuated ATRA‐induced ISG15 conjugation. Knockdown of ISG15 in NB4 APL cells inhibited ISGylation and also attenuated ATRA‐induced differentiation. In summary, we demonstrate the functional importance of UBE2L6 in ATRA‐induced neutrophil differentiation of APL cells and propose that this may be mediated by its catalytic role in ISGylation.

## Graphical abstract

Knockdown of UBE2L6 or ISG15 in NB4 APL cells inhibits ISGylation of protein targets and attenuates ATRA‐induced differentiation.

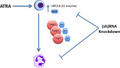

**Abbreviations**
AML: acute myeloid leukemia
APL: acute promyelocytic leukemia
ATRA: all‐trans‐retinoic acid
CML: chronic myeloid leukemia
HERC5: HECT and RLD domain containing E3 ubiquitin protein ligase 5
ISG15: interferon‐stimulated gene 15
NBT: nitro blue tetrazolium
RARα: retinoic acid receptor alpha
UBE1L: ubiquitin‐like modifier‐activating enzyme 7
UBE2L6: ubiquitin/ISG15-conjugating enzyme E2L6
UBLs: ubiquitin‐like modifiers
USP18: ubiquitin‐specific peptidase 18

## 1 Introduction

Acute myeloid leukemia (AML) is a clonal disorder characterized by the accumulation of immature hematopoietic precursors in the bone marrow and peripheral circulation (Caceres‐Cortes, 2013). Overall survival is poor, particularly for patients over 60 years of age, who have an overall 5‐year survival of ~ 10% (Kantarjian and O'Brien, 2010; Thein *et al.*, 2013). Improved therapeutic strategies with tolerable toxicity profiles are needed.

Acute promyelocytic leukemia (APL) is a clinically, pathologically, and molecularly distinct subtype of AML (Tallman and Altman, 2009). It is distinguished in 95% of cases by a translocation of chromosomes 15 and 17, which leads to the expression of a fusion oncoprotein PML‐retinoic acid receptor alpha (RARα) (Tallman and Altman, 2009). This protein disrupts functional retinoid signaling in APL cells, repressing gene transcription and halting myeloid maturation at the promyelocyte stage (Tang and Gudas, 2011). Therapeutic doses of all‐*trans*‐retinoic acid (ATRA) reactivate gene transcription and overcome this differentiation block allowing clinical remission (Tang and Gudas, 2011). As we have previously reviewed, ATRA also encourages the degradation of the PML‐RARα protein through cooperating pathways including proteasomal degradation and autophagy (Orfali *et al.*, 2014).

Cellular protein activity and stability is regulated by post‐translational modification (Krishna and Wold, 1993). One such modification is ‘ubiquitination’, the reversible addition of ubiquitin protein (8.5kDa) to the lysine residues of target substrates (Komander and Rape, 2012). This is catalyzed by a series of enzymes: (a) Ubiquitin‐activating enzymes (E1) use ATP to convert ubiquitin to a high‐energy thioester; (b) ubiquitin‐conjugating enzymes (E2) bind active ubiquitin on their cysteine residues; and (c) ubiquitin‐ligase enzymes (E3) interact with E2 enzymes and catalyze the formation of a covalent bond between ubiquitin and its target substrate. E3 ligases regulate substrate specificity. Ubiquitin can also be removed from target proteins through the action of deubiquitinases (Friend *et al.*, 2014). Ubiquitin‐like modifiers (UBLs) are proteins that share significant structural and some sequence homology with ubiquitin and modify substrates in a similar enzyme‐controlled fashion (Hochstrasser, 2009). While protein ubiquitination is known to alter protein activity or target substrates for degradation by the 26S proteasome, the functional roles of UBL modifications are less well‐defined and remain under investigation (Hochstrasser, 2009). We now recognize at least ten UBLs, including small ubiquitin‐like modifier, autophagy‐related proteins 8 and 12 (autophagy‐related protein 8, autophagy‐related protein 12), and interferon‐stimulated gene 15 (ISG15)—which is an important modification in the present study (Hochstrasser, 2009).

ISG15 is a 15 kDa protein, which contains 2 UBL domains that share 33% and 32% homology with ubiquitin (Sgorbissa and Brancolini, 2012). The conjugation of ISG15 to substrate lysine residues, known as ISGylation, relies on a narrow range of E1, E2, and E3 enzymes (Fig. S1). Ubiquitin‐like modifier‐activating enzyme 7 (UBA7/UBE1L) is the E1 enzyme of ISGylation (Jeon *et al.*, 2010). Ubiquitin/ISG15‐conjugating enzyme E2 L6 (UBE2L6) operates as an E2 enzyme for both ISGylation and ubiquitination (Jeon *et al.*, 2010). Three known E3 ligases determine ISGylation targets, over 300 of which have been proposed (Jeon *et al.*, 2010; Sgorbissa and Brancolini, 2012). HECT and RLD domain containing E3 ubiquitin protein ligase 5 (HERC5) is the dominant E3 for ISGylation and associates with ribosomes to target newly synthesized proteins in a nonspecific manner (Durfee *et al.*, 2010). Tripartite motif containing 25 and ariadne RBR E3 ubiquitin protein ligase 1 show specificity for 14‐3‐3 and 4EHP, respectively (Sgorbissa and Brancolini, 2012). Ubiquitin‐specific peptidase 18 (USP18) removes ISG15 from its substrates and thus negatively regulates the ISGylation pathway (Malakhov *et al.*, 2002). The precise functions of ISGylation remain under investigation and may be contextual. All components of the system are induced on type I interferon stimulation suggesting a functional role in antiviral responses (Sgorbissa and Brancolini, 2012). Free ISG15 has interferon‐stimulating cytokine activity when secreted outside of the cell (Bogunovic *et al.*, 2013).

*ISG15* expression and ISGylation are induced during erythropoiesis, and primary erythroblasts harvested from *ISG15^−/−^* knockout mice show impaired differentiation in *ex vivo* culture (Maragno *et al.*, 2011). Transcriptional profiling of human granulopoiesis has shown that *ISG15* expression is similarly induced during terminal neutrophil differentiation and a PU.1 binding site has been identified within the *ISG15* promoter region (Meraro *et al.*, 2002; Theilgaard‐Monch *et al.*, 2005). To date, however, a functional role for ISGylation in granulopoiesis has not been proven.

Our work has found that *UBE2L6*, the gene encoding the E2 enzyme of ISGylation, is strongly upregulated following ATRA treatment of APL cells. Through a series of short hairpin (sh)RNA knockdown experiments, we have investigated for the first time the functional importance of this enzyme in the ATRA‐mediated granulocytic differentiation of APL cells. We report that inhibiting UBE2L6 expression results in reduced ISGylation and impaired APL cell differentiation. Interference with *ISG15* expression similarly impedes differentiation. Through improving our understanding of ISGylation and protein PTMs involved in ATRA‐mediated differentiation of APL cells, we hope to identify ways of promoting differentiation therapy in other AML subtypes.

## 2 Materials and methods

### 2.1 Cell lines and culture conditions

The human APL cell line NB4 and its ATRA‐resistant NB4R counterpart were kindly gifted by B.E. Torbett and P. Paolo‐Pandolfi, respectively. ATRA‐sensitive human M2 AML HL60 cells were obtained from the Deutsche Sammlung von Mikroorganismen und Zellkulturen (DSMZ, Braunschweig, Germany). Their ATRA‐resistant subclone, HL60R cells were kindly gifted by M. Tschan. All cell lines were maintained in RPMI 1640 (Sigma R8758, Sigma‐Aldrich, Merck, NJ, USA) medium supplemented with 10% fetal calf serum (Sigma F7524) and 1% penicillin/streptomycin (Gibco 15070‐063, ThermoFisher Scientific, Waltham, MA, USA) in a humidified atmosphere containing 5% CO_2_ at 37 °C. For differentiation experiments, cells were seeded at 0.2 × 10^5^ cells per mL and treated for 4 days with 1 μm ATRA (Sigma R2625) diluted from a 1 mm stock in 100% EtOH.

### 2.2 Patient study

A cohort of 98 AML patient samples, collected through the HOVON/SAKK (Dutch‐Belgian Hematology‐Oncology/Swiss Group for Clinical Cancer Research Cooperative Group) protocols 04, 04A, 29, and 42 between 1987 and 2006, were provided by P. Valk and B. Lowenberg. Patient characteristics have been previously outlined (Schlafli *et al.*, 2012). Primary neutrophils from healthy donors were isolated using Polymorphprep (Axis‐Shield, Dundee, Scotland). All patients provided written informed consent in accordance with the Declaration of Helsinki.

### 2.3 RNA extraction, quantitative real‐time PCR (qPCR), TaqMan low‐density array

Total cellular RNA was harvested using TRIzol (Invitrogen 15596‐018, ThermoFisher Scientific, Waltham, MA, USA), according to the manufacturer’s protocol. 1μg of RNA was reverse‐transcribed using qScript (Quanta Biosciences #95047, Beverly, MA, USA) as per product protocol at a final reaction volume of 20 μL, and the resulting cDNA was diluted 1 : 10 in H_2_0. Subsequent qRT–PCRs were carried out using 2 μL of template together with 1× SYBR Green Supermix (Quanta Biosciences #84091), forward and reverse primers at 0.25 µm and 2.5 μL H_2_0 in a final reaction volume of 15 μL. Reactions were run on a Bio‐Rad MyiQ™ (Irvine, CA, USA) Single Color Real‐time PCR detection system with each cycle including a 94 °C × 20 s denaturation step, 60 °C × 20 s annealing step, and a 72 °C x 30 s extension step. Primer pairs were designed to span distinct exon software to avoid genomic DNA signaling, and gene expression amplicons were validated with sequencing at the Genomics Resources Core Facility, WCMC. Sequences of specific primers were as follows: UBE2L6_F CTGGAAGCCTTGCACCAAGA, UBE2L6_R GAACATGAGTTAGGAGGGCCG, ISG15_F GGTGGACAAATGCGACGAAC, and ISG15_R TCGAAGGTCAGCCAGAACAG. The transcript levels in biological replicates (*n* = 3) were normalized to *hPRT* transcript levels, and relative differences were calculated using the Pfaffl method. Graphical displays and measurements of statistical significance were performed on graphpad prism software (San Diego, CA, USA).

### 2.4 Lentiviral shRNA transduction

pLKO.1 lentiviral vectors expressing small hairpin shRNAs targeting both UBE2L6 and ISG15 were purchased from Sigma‐Aldrich along with a nontargeting shRNA control (SCH002) in bacterial glycerol stocks. For each gene, five shRNAs were initially tested for efficiency by measuring mRNA levels by qPCR and two shRNAs were then selected for use in further experiments. (shUBE2L6_499 = NM_004223.3‐499s1c1/ TRCN0000007284, shUBE2L6_1082 = NM_004223.3‐1082s1c1/ TRCN0000007281, shISG15_319 = NM_005101.3‐319s21c1/ TRCN0000237825, and shISG15_352 = NM_005101.3‐352s21c1/ TRCN0000237824). Lentiviral production and transduction was performed as previously described (Tschan *et al.*, 2003). All vectors contain a puromycin resistance gene, and transduced cell clones were selected for 4 days using 1.5 µg·mL^−1^ puromycin.

### 2.5 Morphology examination

Cells were cytospun onto glass slides and stained with Rapi‐Diff (Braidwood Laboratories 22007, 22008, 22009, London, UK) according to product guidelines. Morphology was examined using an Olympus DP70 digital microscope at 400X magnification (Mason Technology, Cork, Ireland).

### 2.6 Nitro Blue Tetrazolium (NBT) assay

Cells were incubated with 0.2% nitro blue tetrazolium (Sigma N5514) and 40 ng·mL^−1^ phorbol 12‐myristate 13‐acetate (PMA) (Sigma P8139) in RPMI for 20 min at 37 °C, washed, and cytospun onto glass slides on a base of PBS/1% FBS. Slides were then counterstained with 0.5% safranin O (Sigma 84120) in 20% EtOH and coverslipped with Entellan (Merck 1076910100, Branchburg, NJ, USA). Nitro blue tetrazolium‐positive cells were then examined, counted in triplicate, and presented accordingly.

### 2.7 Western blotting

Cellular protein extracts were lysed in modified RIPA buffer (50 mm Tris/HCl—pH 7.4, 150 mm NaCl, 0.25% sodium deoxycholate, 1% Igepal, 1 mm EDTA, 1× Pefabloc, 1× protease inhibitor cocktail, 1 mm Na_3_VO_4_, 1 mm NaF). Protein samples were separated on NuPAGE 4–12%, Bis/Tris gels (Invitrogen NP0322), and electrophoretically transferred onto PVDF membranes (Invitrogen IB401001). Primary antibodies were as follows: anti‐UBE2L6 (Abgent AP2118A, San Diego, CA, USA), anti‐ISG15 (Proteintech 15981‐1‐AP, Manchester, UK), and anti‐β‐actin (Sigma A5441). Proteins were visualized using relevant IR‐DYE secondary antibodies and quantified on the Odyssey IR imaging system (Li‐Cor, Cambridge, UK).

### 2.8 Flow cytometry

Live cells were incubated for 30 min with PE‐conjugated anti‐CD11b antibody (eBioscience 12‐0118 or Immunotools #21279114, San Diego CA, USA) in 1% albumin/ PBS, and washed with PBS prior to analysis. Fluorescence was detected using a BD‐LSRII flow cytometer (BD Biosciences, Oxford, UK). Data analysis and histogram overlays were performed on flowjo software (FlowJo, Becton, Dickinson & Company, Franklin Lakes, NJ, USA).

## 3 Results

### 3.1 UBE2L6 is induced during the neutrophil differentiation of leukemic cells

We have previously examined the gene expression changes induced by ATRA in APL cells by sequencing RNA extracted from NB4 cells treated with 1 μm ATRA for 72 h alongside untreated controls (Orfali *et al.*, 2019). These data showed that ATRA induced a 200‐fold increase in *UBE2L6* expression. Other members of the ISGylation pathway were also found to be coregulated (RNAseq data reproduced in Table 1). As NB4 cells respond to ATRA by differentiating toward mature neutrophils, this prompted us to question whether UBE2L6 expression is important for leukemic cell differentiation.

**Table 1:** ATRA‐induced expression changes in ISGylation genes.

Gene	Name	Fold change in expression
*UBE2L6*	Ubiquitin/ISG15‐conjugating enzyme E2L6	200.93
*ISG15*	Interferon‐stimulated gene 15	17.54
*USP18*	Ubiquitin‐specific peptidase 18	12.53
*UBE1L*	Ubiquitin‐like modifier‐activating enzyme 7	7.77
*HERC5*	HECT and RLD domain containing E3 ubiquitin protein ligase 5	3.62
*TRIM25*	Tripartite motif containing 25	2.16

**Figure 1:**
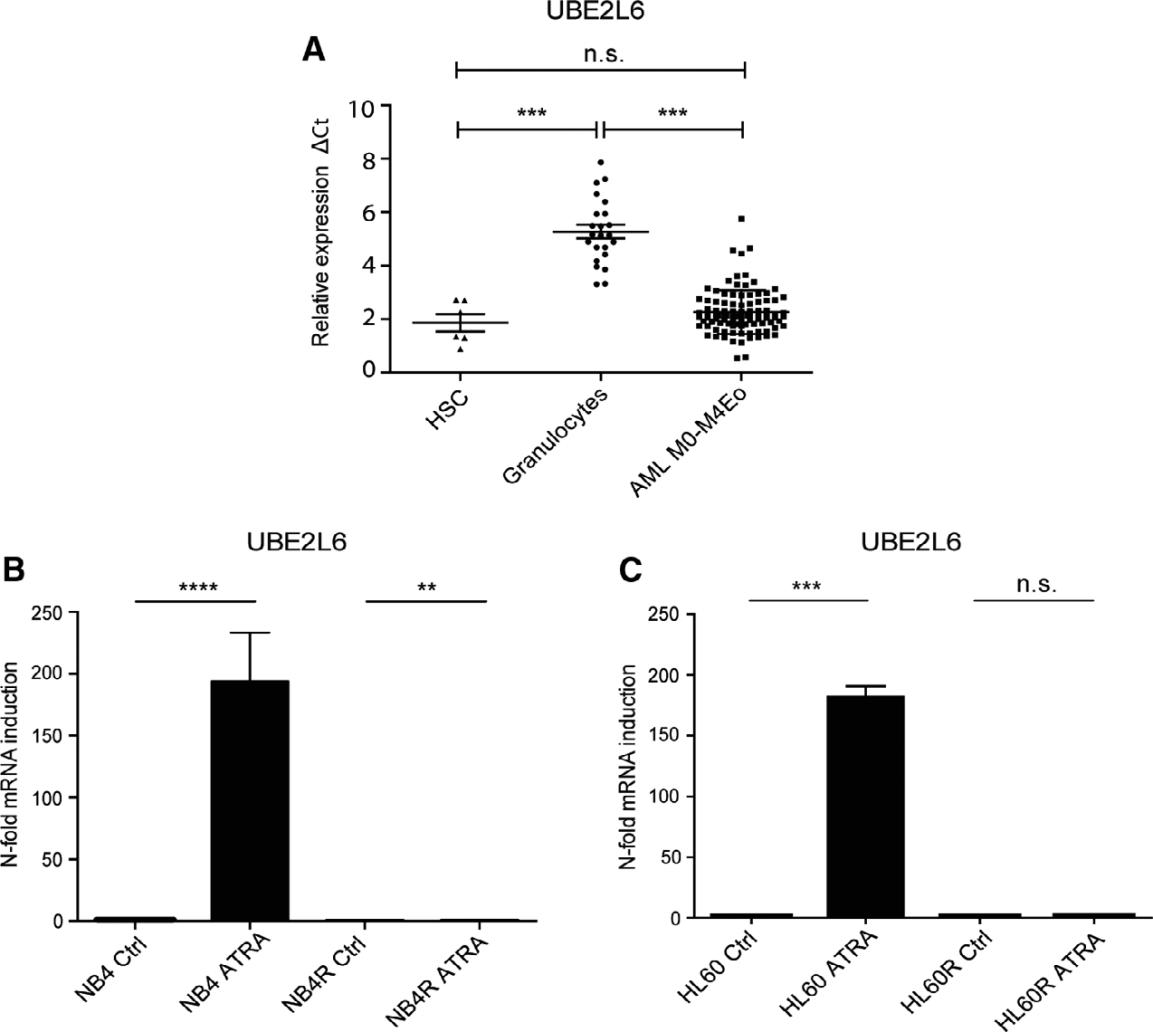
*UBE2L6* expression is increased during leukemic cell differentiation. (A) *UBE2L6* mRNA levels of primary AML patient samples, normal CD34^+^(HSC) cells, and mature granulocytes from healthy donors were quantified using qPCR. The relative ΔCt expression was calculated by the difference in *UBE2L6* expression to the housekeeping genes *HMBS* and *ABL* (Mann–Whitney *U*‐test ****P ≤ 0.0001). (B) NB4 and NB4R cells were seeded at 0.2 × 10^5^ cells per mL and treated with 1 μm ATRA for 72 h. Successful differentiation was confirmed in NB4 cells by flow cytometric analysis of CD11b expression. ATRA‐resistant NB4R cells did not differentiate (data not shown). Total RNA was extracted, and *UBE2L6* mRNA expression was quantified by qPCR. Values are given as *n*‐fold induction compared with untreated cells and normalized to housekeeping gene *hPRT* (*n* = 3) (*t*‐test ****P ≤ 0.0001, **P ≤ 0.01). (C) HL60 and HL60R cells were treated with 1 μm ATRA for 96 h. Successful HL60 differentiation was confirmed by qPCR measurement of GCSFR expression. ATRA‐resistant HL60R cells failed to differentiate (data not shown). Total RNA was extracted, and *UBE2L6* expression was quantified by qPCR. Values are given as *n*‐fold induction compared with untreated cells and normalized to housekeeping gene *HMBS* (*n* = 3) (*t*‐test ***P ≤ 0.001).

We first examined *UBE2L6* mRNA expression in 98 primary AML patient samples (M0–M4), six samples of normal CD34^+^ (HSC) cells, and 24 donated mature granulocyte samples using a TaqMan low‐density array. Relative *UBE2L6* mRNA levels are shown as differences in Ct values as compared to mRNA levels for the housekeeping genes *HMBS* and *ABL1*. Expression was significantly lower in AML patient samples and HSC cells than in granulocytes, suggesting that increased expression may be important for the mature granulocyte phenotype (Mann–Whitney *U*‐test, ****P ≤ 0.0001) (Fig. 1A).

To test this hypothesis further, we treated NB4 cells with ATRA along with their ATRA‐resistant counterparts NB4R cells. We measured *UBE2L6* expression by quantitative real‐time (q)PCR at 72 h, assessing Ct values relative to the housekeeping gene *hPRT*. Validating our earlier RNA sequencing observations, we detected a 180‐fold increase in *UBE2L6* expression in differentiating NB4 cells (****P ≤ 0.0001), but only a 0.23‐fold difference in NB4R cells (**P = 0.0021) (Fig. 1B). The HL60 cell line (human M2 AML), although it does not carry the PML‐RARα oncoprotein, also differentiates down a granulocytic lineage in response to ATRA therapy and can be used as a second model of leukemic cell differentiation. Following 96 h of ATRA treatment, we found a 189‐fold increase in *UBE2L6* expression in HL60 cells (***P = 0.0003). ATRA‐resistant HL60R cells, however, failed to induce *UBE2L6* (Fig. 1C).

These results indicate that *UBE2L6* is prominently upregulated during leukemic cell differentiation rather than solely on ATRA treatment and that this effect is not restricted to APL cells carrying the PML‐RARα fusion oncoprotein.

### 3.2 Knockdown of UBE2L6 inhibits ATRA‐induced neutrophil differentiation of NB4 APL cells

**Figure 2:**
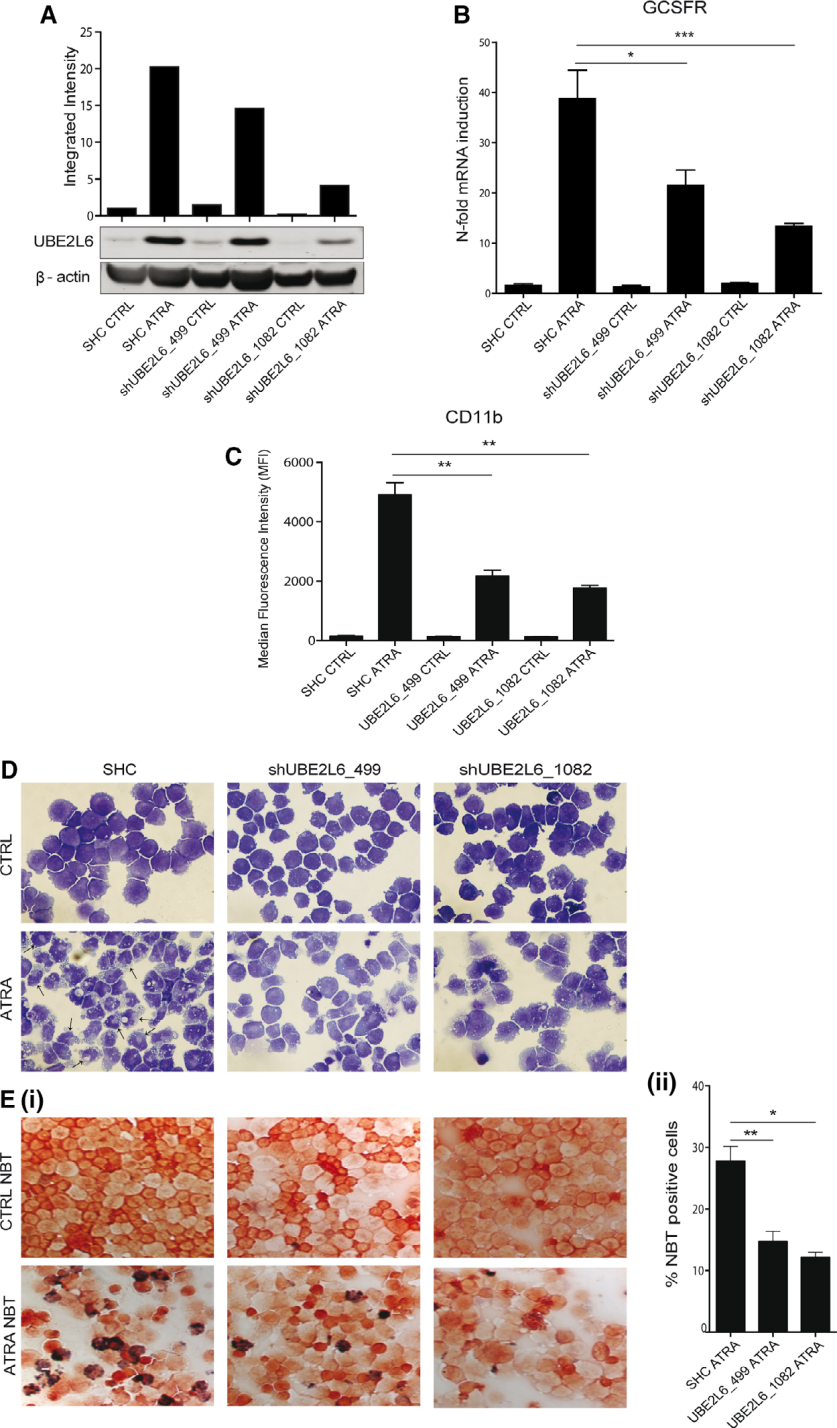
*UBE2L6* inhibition attenuates APL cell differentiation. NB4 cells expressing nontargeting shRNA (SHC) or shRNA targeting *UBE2L6* (shUBE2L6_499 and shUBE2L6_1082) were seeded at 0.2 × 10^5^ cells per mL and treated for 72 h with 1 μm ATRA. (A) Functional knockdown efficiency was tested by measuring UBE2L6 protein levels in whole‐cell lysates by immunoblot at 72 h. β‐actin was used as a loading control. (B) Total RNA was extracted, and differentiation was assessed by measuring *GCSFR* mRNA expression by qPCR. Values are given as *n*‐fold induction compared with untreated cells and normalized to housekeeping gene *HMBS* (*n* = 3) (*t*‐test ***P ≤ 0.001, *P ≤ 0.05). (C) Surface CD11b protein expression on live cells was measured by flow cytometry as a second assay of differentiation. Median fluorescence intensities (MFIs) are shown at 72 h (*n* = 3) (*t*‐test **P ≤ 0.01). (D) Morphologic appearance of treated cells at 72 h. Neutrophil differentiation evidenced by increased cytoplasmic volume and nuclear lobulation, indicated with arrows. (E) Neutrophil function was tested using nitro blue tetrazolium at 72 h (i) Differentiated cells reduce nitro blue tetrazolium to a blue color. (ii) nitro blue tetrazolium ‐positive cells were counted in triplicate and presented as mean ± SEM (*t*‐test **P ≤ 0.01, *P ≤ 0.05) (magnification 400×).

In order to investigate whether UBE2L6 has a functional role in leukemic cell differentiation, we generated *UBE2L6* knockdown NB4 cells using a lentiviral delivery system to deliver target‐specific shRNA. NB4 cells transduced with a nontargeting shRNA were used as a control (SHC). Functional knockdown was confirmed by detecting reduced UBE2L6 protein levels following ATRA treatment in two knockdown clones: shUBE2L6_499 and shUBE2L6_1082. Superior knockdown efficiency is evident in shUBE2L6_1082 (Fig. 2A).

ATRA‐induced neutrophil differentiation was reduced in *UBE2L6* knockdown NB4 clones compared with control cells. At a transcript level, we detected reduced expression of granulocyte colony‐stimulating factor receptor (*GCSFR*), a marker of neutrophil differentiation, by qPCR at 72 h (*P = 0.0123, ***P = 0.0004) (Fig. 2B). At a protein level, surface CD11b expression was reduced in ATRA‐treated knockdown cells when analyzed by flow cytometry at 72 h. A direct correlation was observed between UBE2L6 knockdown efficiency and detectable CD11b levels, with both shUBE2L6_499 and shUBE2L6_1082 showing a significant reduction in CD11b (**P = 0.0017 and *P* = 0.0067, respectively) (Fig. 2C). Morphologically, ATRA‐treated control cells displayed characteristic features of granulocytic differentiation with increased cytoplasmic volume and visible nuclear indentation (Fig. 2D lower left panel, arrows). This phenotype was stunted in knockdown clones (Fig. 2D lower middle and right panels). Finally, we assessed functional differentiation using the nitro blue tetrazolium assay, which tests the reducing power of the neutrophil enzyme alkaline phosphatase. We observed and quantified a decreased nitro blue tetrazolium reduction in UBE2L6 knockdown clones (**P = 0.0069, *P = 0.0354), with a direct correlation again seen between knockdown efficiency and functional differentiation (Fig. 2E (i) lower panels and Fig. 2E (ii)).

Together, these results demonstrate that UBE2L6 depletion impedes ATRA‐mediated granulocytic differentiation of APL cells and prompted us to question the potential mechanism involved in this differentiation block.

### 3.3 UBE2L6 mediates protein ISGylation in ATRA‐treated APL cells

**Figure 3:**
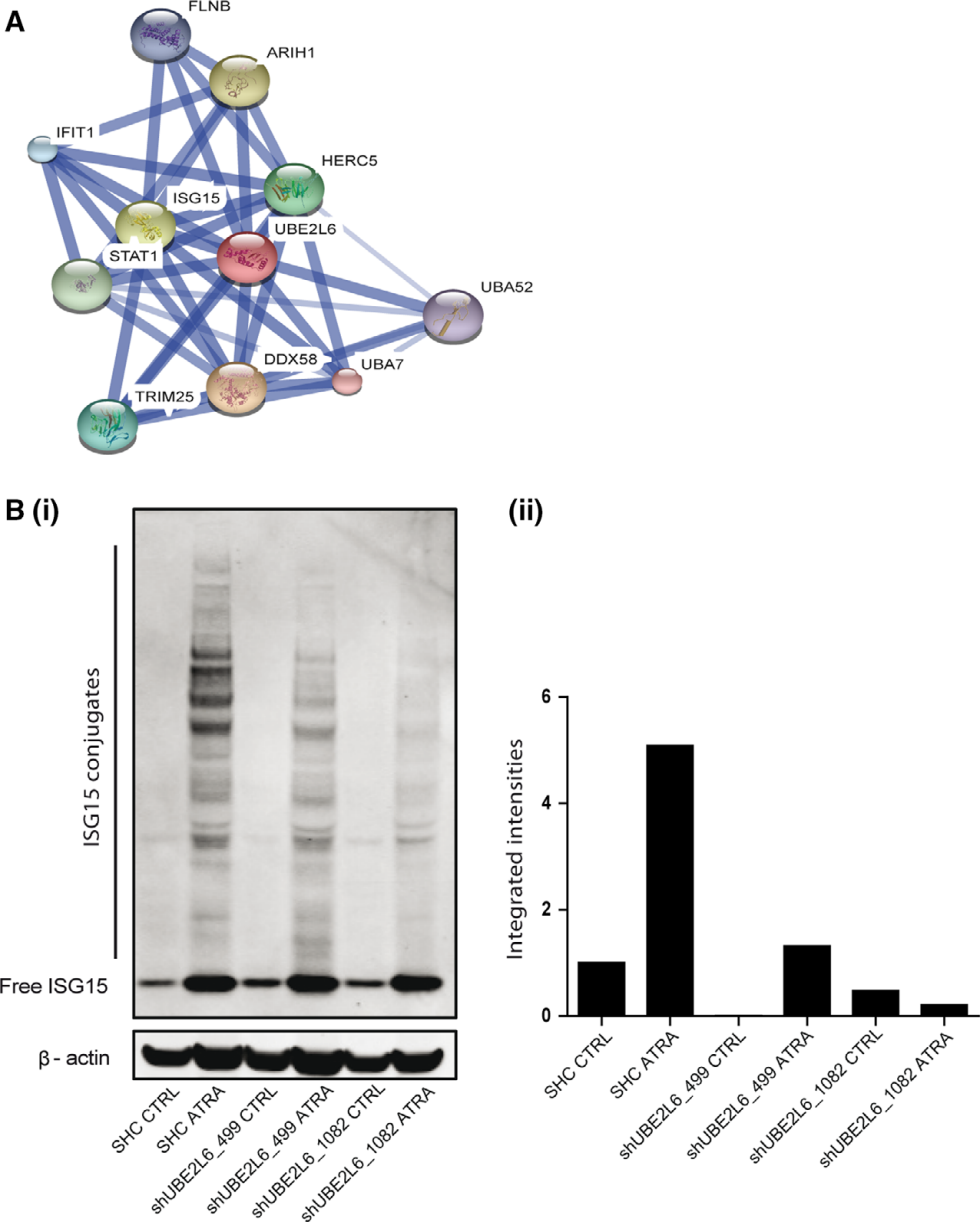
UBE2L6 regulates ATRA‐induced ISGylation. (A) Proteins known with a high confidence to interact with UBE2L6 are shown. Image created using the STRING proteomics database (http://www.string-db.org). (B) (i) Levels of free and conjugated ISG15 were measured by immunoblot in whole‐cell lysates extracted from NB4 cells expressing either nontargeting shRNA (SHC) or shRNA targeting UBE2L6 (shUBE2L6_499 and shUBE2L6_1082) following a 72 h of treatment with 1 μm ATRA. β‐actin was used as a loading control. (ii) Conjugated ISG15 levels were normalised to β‐actin and presented as integrated intensities.

As described earlier, UBE2L6 is an E2 ligase critical in the conjugation of ISG15 to target proteins during ISGylation, a process with an unknown role in leukemic cell differentiation. A search of UBE2L6 human protein interactions on the publicly available STRING database (*S*earch *T*ool for the *R*etrieval of *In*teracting *G*enes/Proteins) (http://www.string-db.org) (Franceschini *et al.*, 2013) depicts a high confidence of interaction between UBE2L6 and ISG15, as well as interactions with the E1 and all E3 ligases of the ISGylation pathway (Fig. 3A).

We tested levels of both free and conjugated ISG15 proteins in *UBE2L6* knockdown NB4 cells using western blot analysis. We found a prominent induction of free ISG15 protein following ATRA treatment in control cells and in knockdown clones. Notably, conjugated ISG15 was markedly increased after 72 h of ATRA treatment in control cells but was impaired in *UBE2L6* knockdown cells, with the most efficient knockdown clone shUBE2L6_1082 having the least amount of visible conjugates (Fig. 3B (i,ii)).

### 3.4 ISG15 is induced during the neutrophil differentiation of leukemic cells

**Figure 4:**
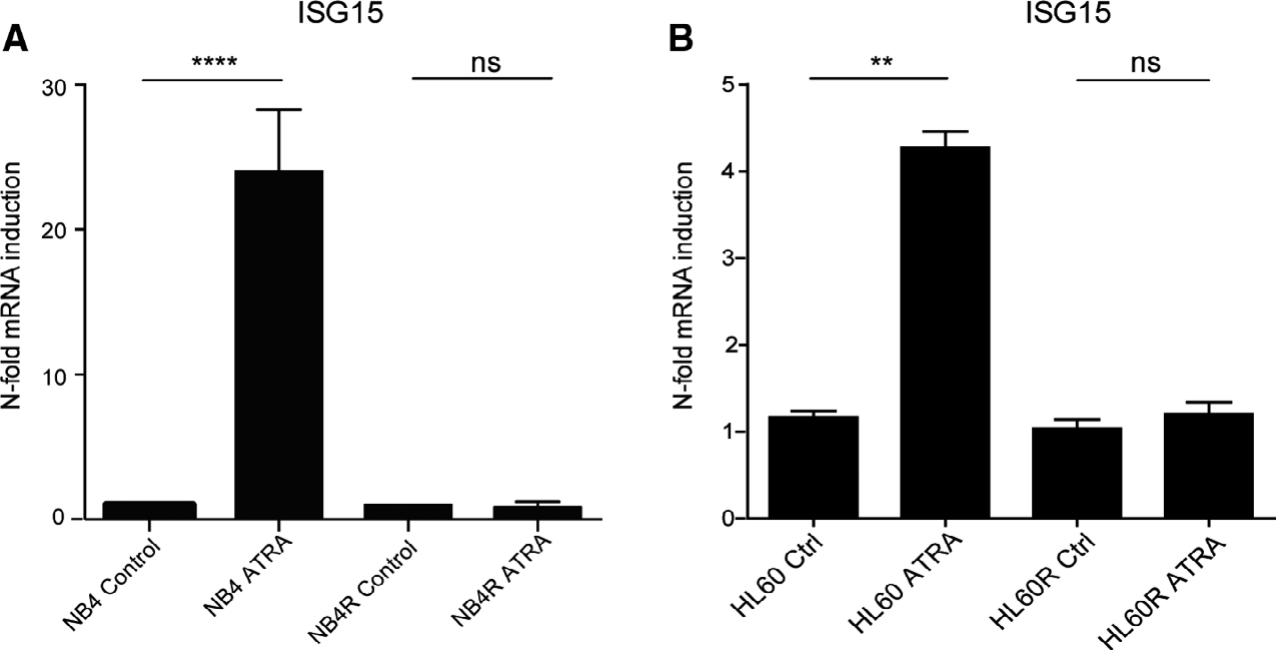
ISG15 expression is induced during leukemic cell differentiation. (A) NB4 and NB4R cells were seeded at 0.2 × 10^5^ cells per mL and treated with 1 μm ATRA for 72 h. Successful differentiation was confirmed in NB4 cells by flow cytometric analysis of CD11b expression. ATRA‐resistant NB4R cells did not differentiate (data not shown). Total RNA was extracted, and *ISG15* mRNA expression was quantified by qPCR. Values are given as *n*‐fold induction compared with untreated cells and normalized to housekeeping gene *hPRT* (*n* = 3) (*t*‐test ****P ≤ 0.0001). (B) HL60 and HL60R cells were treated with 1 μm ATRA for 96 h. Successful HL60 differentiation was confirmed by qPCR measurement of GCSFR expression. ATRA‐resistant HL60R cells failed to differentiate (data not shown). Total RNA was extracted, and *ISG15* expression was quantified by qPCR. Values are given as *n*‐fold induction compared with untreated cells and normalized to housekeeping gene *HMBS* (*n* = 3) (*t*‐test **P ≤ 0.01).

Our RNA sequencing data showed a 17.54‐fold increase in *ISG15* gene expression in NB4 cells with ATRA treatment (Orfali *et al.*, 2019) (Table 1). We validated this finding by qPCR measurement of *ISG15* expression in ATRA‐treated NB4 cells at 72 h, which showed a 23‐fold increase in expression in differentiating cells (****P ≤ 0.0001). No significant change in expression was found in ATRA‐treated NB4R cells (Fig. 4A). HL60 cells undergoing differentiation with ATRA treatment showed a fourfold induction of ISG15 at 96 h (**P = 0.0011), whereas no induction was seen in HL60R cells (Fig. 4B). These findings associate ISG15 induction with leukemic cell differentiation rather than solely with ATRA treatment.

### 3.5 Knockdown of ISG15 inhibits ATRA‐induced neutrophil differentiation of NB4 APL cells

**Figure 5:**
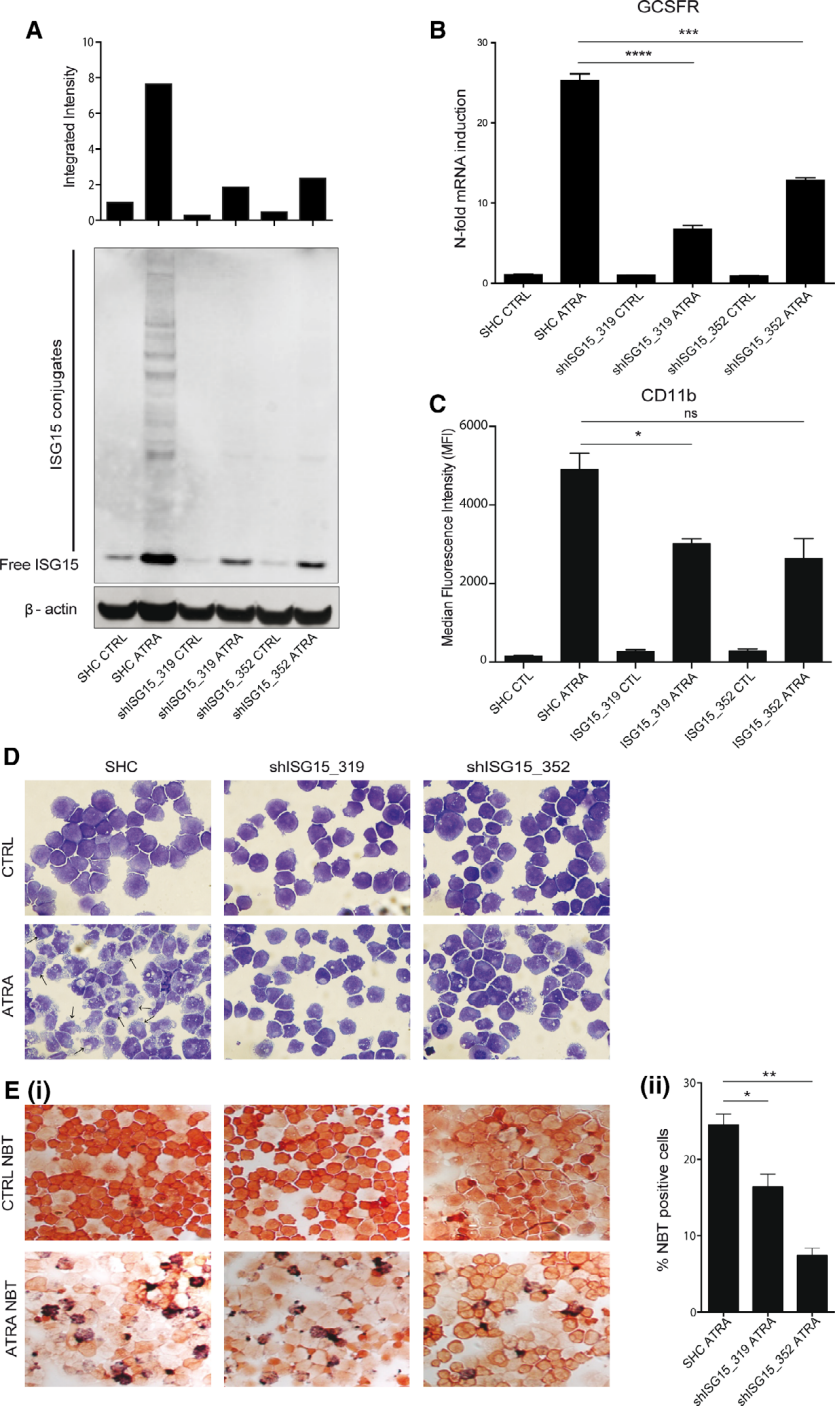
Inhibition of *ISG15* impedes APL cell differentiation. NB4 cells expressing nontargeting shRNA (SHC) or shRNA targeting *ISG15* (shISG15_319 and shISG15_352) were seeded at 0.2 × 10^5^ cells per mL and treated for 72 h with 1 μm ATRA. (A) Functional knockdown efficiency was tested by measuring protein levels of both free and conjugated ISG15 in whole‐cell lysates by immunoblot at 72 h. β‐actin was used as a loading control. (B) Total RNA was extracted, and differentiation was assessed by measuring *GCSFR* mRNA expression by qPCR. Values are given as *n*‐fold induction compared with untreated cells and normalized to housekeeping gene *HMBS* (*n* = 3) (*t*‐test ****P ≤ 0.0001, ***P ≤ 0.001). (C) Surface CD11b protein expression on live cells was measured by flow cytometry as a second assay of differentiation. MFIs are shown at 72 h (*n* = 3) (*t*‐test *P ≤ 0.05). (D) Morphologic appearance of treated cells at 72 h. Neutrophil differentiation evidenced by increased cytoplasmic volume and nuclear lobulation indicated with arrows. (E) Neutrophil function was tested using nitro blue tetrazolium at 72 h. (i) Differentiated cells reduce nitro blue tetrazolium to blue color. (ii) nitro blue tetrazolium‐positive cells were counted in triplicate and presented as mean ± S.E.M (*t*‐test **P ≤ 0.01, *P ≤ 0.05) (magnification 400×).

To investigate whether the induction of ISG15 during ATRA‐mediated leukemic cell differentiation had functional significance, we knocked down *ISG15* in NB4 APL cells. We confirmed efficient ISG15 protein knockdown in two clones, shISG15_319 and shISG15_352, detecting reduced basal levels of free ISG15 in knockdown cells compared with controls and by detecting reduced induction of free ISG15 by 72 h of ATRA treatment. ISG15 conjugates were not seen before or after ATRA treatment in knockdown cells consistent with the knockdown blocking ATRA‐induced ISGylation (Fig. 5A).

ATRA‐induced neutrophil differentiation was reduced in both ISG15 knockdown clones compared with control cells, analogous to UBE2L6‐depleted cells. At the transcript level, we found reduced GCSFR mRNA expression in ATRA‐treated knockdown clones compared with ATRA‐treated control cells at 72 h (****P ≤ 0.0001, **P = 0.0002, respectively) (Fig. 5B). At the protein level, surface CD11b expression was reduced in both ATRA‐treated knockdown clones (*P = 0.0434 in shISG15_319 clone), but did not achieve significance in the shISG15_352 knockdown clone (Fig. 5C). ISG15 knockdown cells failed to morphologically differentiate into mature myeloid forms after 72 h of ATRA treatment (Fig. 5D lower middle and right panels), and their ability to reduce nitro blue tetrazolium was also diminished as is shown in Fig. 5E (i) lower middle and right panels. Decreased nitro blue tetrazolium reduction was quantified and is presented in Fig. 5E (ii) (*P = 0.0312, **P = 0031).

Our findings suggest a functional role for ISGylation in the ATRA‐mediated neutrophil differentiation of APL cells. In the context of our earlier results, ISGylation may be the prominent mechanism by which UBE2L6 regulates differentiation.

## 4 Discussion

Our results demonstrate that *UBE2L6* is underexpressed in AML cells compared with their mature myeloid counterparts. Using two cell line models of AML cell differentiation, we show that cells undergoing ATRA‐mediated neutrophil differentiation strongly induce *UBE2L6*. We show that shRNA depletion of *UBE2L6* in leukemic cells impedes their ability to differentiate, reporting for the first time a functional importance for this enzyme in ATRA‐mediated leukemic cell differentiation.

While UBE2L6 was first identified as an E2‐conjugating enzyme in ubiquitination, it is now thought to preferentially function as an E2 enzyme for ISG15 conjugation (Jeon *et al.*, 2010; Kim *et al.*, 2004; Zhao *et al.*, 2004). We demonstrate that UBE2L6 modulates ISGylation in leukemic cells and further show that genetic inhibition of *ISG15* strongly interferes with the neutrophil differentiation of ATRA‐treated APL cells. We hence propose that the effects of UBE2L6 on leukemic cell differentiation are likely to involve its activity in ISGylation.

In addition to ISG15 and other elements of the cellular ISGylation machinery, *UBE2L6* is induced by type 1 interferon signaling and contains an interferon‐stimulated response element in its promoter region (Kim *et al.*, 2004). ATRA has previously been shown to upregulate ISGylation machinery in NB4 cells and to stimulate ISGylation (Dao *et al.*, 2006). This effect is thought to be due to ATRA stimulating the secretion of type I interferon as antibody‐mediated blockade of the interferon relative receptor complex suppresses this ISGylation (Dao *et al.*, 2006). Experiments by Pitha‐Rowe and colleagues identified that UBE1L, the E1‐activating enzyme of ISGylation, is induced by ATRA treatment in ATRA‐sensitive but not ATRA‐resistant APL cells (Kitareewan *et al.*, 2002). Subsequently, this group identified RARα binding in a domain of the UBE1L promoter, which was repressed by the PML‐RARα oncoprotein (Kitareewan *et al.*, 2002). While our observation that *UBE2L6* is induced only in ATRA‐sensitive NB4 APL cells could be explained by direct repression by PML‐RARα, we observe equivalent UBE2L6 expression levels in ATRA‐sensitive and ATRA‐resistant HL60 cells which do not carry the PML‐RARα oncoprotein. We thus speculate that UBE2L6 is activated during the leukemic cell differentiation program with a functional purpose regardless of the presence of fusion oncoproteins.

Equally, *ISG15* induction has been reported following ATRA treatment only in differentiating NB4 APL cells and not in ATRA‐resistant NB4R cells (Guo *et al.*, 2010; Pitha‐Rowe *et al.*, 2004). Up until now, a similar finding in HL60 and HL60R cells has not been reported. Our findings again suggest that ISG15 is activated during the cellular differentiation program rather than being regulated by the PML‐RARα oncoprotein. The ISG15 promoter contains a PU.1 binding site in addition to two ISREs, and it is possible that it is activated by this transcription factor during leukemic cell differentiation (Meraro *et al.*, 2002).

The precise cellular functions of ISGylation remain under speculation. Proteomic studies in a range of cell lines have now collectively identified over 300 putative ISGylation targets, with no functional or compartmental class over‐represented among them (Giannakopoulos *et al.*, 2005; Malakhov *et al.*, 2003; Zhao *et al.*, 2005). HERC5, the primary E3 ligase of ISGylation, associates with ribosomes and broadly captures newly synthesized proteins of both endogenous origin and exogenous origin for ISGylation (Durfee *et al.*, 2010). Induced by both type I interferon and lipopolysaccharide, ISGylation is thought to play a role in our defense against viral pathogens, but the exact mechanisms at play remain under investigation (Zhang and Zhang, 2011). While ubiquitination can modulate protein function or promote proteasomal degradation (depending on specific linkages) (Ebner and Versteeg, 2017), it is unclear whether ISGylation has analogous effects on target proteins and whether these effects may be contextual (Zhang and Zhang, 2011). ISGylation can stabilize proteins, competitively preventing their ubiquitination and subsequent degradation, as is seen with interferon regulatory factor 3 (Shi *et al.*, 2010). It may also modify enzymes involved in ubiquitination impeding their function and thus negatively regulating proteasomal degradation of ubiquitin substrates (Takeuchi and Yokosawa, 2005; Zou *et al.*, 2005). ISGylation might also inactivate or destabilize proteins through proteasomal channels. UBE1L‐mediated ISGylation of cyclin D1 in lung cancer cells reduces detectable protein levels with an antiproliferative effect. The reduction in cyclin D1 is reversed with the overexpression of the deISGylating enzyme USP18 (Feng *et al.*, 2008). Further study is warranted into the context‐dependent and possibly tumor‐suppressive actions of ISGylation in physiologic and pathologic settings.

An inhibitory effect on ATRA‐mediated neutrophil differentiation of NB4 APL cells as a direct result of shRNA depletion of either *UBE2L6* or *ISG15* has not been previously reported. Our findings propose a functional role for these genes in differentiation. We have previously reviewed the effects of ATRA on the PML‐RARα protein in APL cells (Orfali *et al.*, 2014). Briefly, in addition to derepressing transcription, ATRA induces the degradation of the PML‐RARα oncoprotein through caspase‐3‐mediated cleavage, ubiquitin/proteasome‐mediated proteolysis, and lysosomal‐mediated autophagy (Orfali *et al.*, 2014). Investigating a temporal correlation between the reduction in PML‐RARα and an induction of UBE1L in ATRA‐treated NB4 cells, Shah *et al. *(2008) reported ISGylation of the PML domain of PML‐RARα with subsequent repression. This repression was opposed by the overexpression of USP18. Subsequent work showed that knockdown of USP18 destabilized PML‐RARα and promoted apoptosis in NB4 APL cells but did not have an effect on differentiation (Guo *et al.*, 2010). A similar destabilizing effect of ISGylation on a leukemic oncoprotein is suggested for the BCR‐ABL kinase that drives chronic myeloid leukemia (CML). The expression of BCR‐ABL in mouse bone marrow cells results in splenomegaly and an abnormal myeloproliferation resembling CML. Bone marrow cells harvested from USP18^−/−^ mice and transfected with BCR‐ABL prior to transplantation into wild‐type recipient mice developed a CML‐like state in only 40% of cases, whereas all mice transplanted with USP18^+/+^ BCR‐ABL expressing cells developed disease (Yan *et al.*, 2007). Degradation of PML‐RARα may be one mechanism through which UBE2L6 and ISGylation contribute to ATRA‐mediated APL cell differentiation as we have observed. However, we speculate that this pathway may have broader functions in this process. This is supported by a proposed role for ISGylation machinery in the differentiation of other hemopoietic cells. High levels of USP18 in murine hematopoietic cells block the cytokine‐induced terminal differentiation of monocytic cells (Liu *et al.*, 1999). ISG15 along with UBE1L and UBCM8 (the murine orthologue of UBE2L6) is induced during erythroid development in mice, and erythroblasts cultured *ex vivo* from ISG15^−/−^ mice show impaired differentiation (Maragno *et al.*, 2011). Future work will examine the effects of modulating ISGylation in non‐APL models of leukemic differentiation.

Our previous work has proposed that promoting autophagy may enhance the differentiating effects of ATRA on leukemic cells (Orfali *et al.*, 2015). With further study, promoting ISGylation may prove to have similar benefits. Inhibiting USP18, the negative regulator of ISGylation, has been shown in both cell line and *in vivo* systems to enhance ISG15 conjugation (Ketscher *et al.*, 2015). A small‐molecule inhibitor of this isopeptidase is awaited and will greatly advance study in this arena (Basters *et al.*, 2012).

## 5 Conclusions

We have identified a novel function of UBE2L6 in the granulocytic differentiation of APL cells, mediated by ISGylation. Our work contributes to the growing area of study of post‐translational protein alteration by ubiquitin‐like modifiers. A greater understanding of the protein handling that occurs during leukemic cell differentiation might allow us to modulate these processes and broaden the application of differentiation therapy for the improved treatment of AML.

## Conflict of interest

All authors declare no conflict of interest.

## Author contributions

NO, DS‐K, TRO’D, NPM, LJG, MRC, MPT, and SLM conceived, designed, and conducted the experiments; interpreted the results; and wrote the manuscript.

## Supporting information


**Fig. S1** **. **ISGylation—in the first step of ISGylation, ISG15 is activated by the E1 enzyme UBE1L in an ATP‐dependent reaction.
