# Supplementary figures and images for: Inhibition of UBE2L6 attenuates ISGylation and impedes ATRA‐induced differentiation of leukemic cells
Source: Mol Oncol. 2020 May 1;14(6):1297–309. doi: 10.1002/1878-0261.12614 (PMC7266268; doi:10.1002/1878-0261.12614)

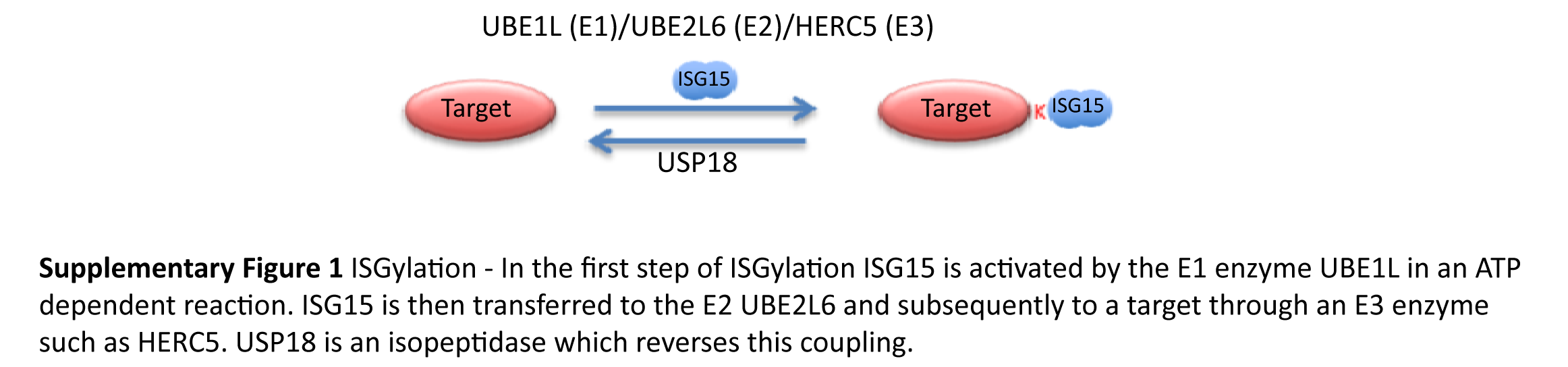

Supplement: Supplementary file 1 — Fig. S1 . ISGylation—in the first step of ISGylation, ISG15 is activated by the E1 enzyme UBE1L in an ATP‐dependent reaction. [file MOL2-14-1297-s001.tif]
